# 

**Published:** 1907-10-15

**Authors:** 


### ANNOUNCEMENTS.

New Board of Examiners for Michigan.—The Governor has appointed under the new law Drs. C. H. Oakman, of Detroit for five years, Dr. A. W. Haidle, of Negaunee, for four years and Dr. A. D. Robinson, of Grand, Rapids, for three years. Dr. A. L. LeGro of Detroit holds over for one year and Dr. E. A. Honey of Kalamazoo for two years. Dr. Oakman is president of the board and Dr. Honey is secretary and treasurer. The new law seems to be favorably commented on, and the new board is busy registering present legal practitioners.

National Dental Examiners' Association.——Elected the following officers: President, Frank O. Hetrick, Ottawa, Kan.; vice-presidents, F. A. Shotwell, Rogersville, Tenn.; F. R. Henshaw, Middletown, Ind.; J. J. Wright, Milwaukee, Wis.; Charles A. Meeker, Newark, N. J., was re-elected secretary-treasurer, which position he has filled for the past fourteen years.

NATIONAL ASSOCIATION OF DENTAL FACULTIES.—Elected the following officers; President, M. W. Foster, Baltimore; vice-president, H. B. Tileston, Louisville; secretary, G. E. Hunt, Indianapolis; treasurer, H. R. Jewett, Atlanta.

THE NATIONAL DENTAL ASSOCIATION'S OFFICERS, 1907-08.—The National Dental Association, at its eleventh annual session, Minneapolis, July 31st, elected the following officers for the ensuing year: President, Wm. Carr, New York City; vice-president for the east, Wilbur F. Litch, Philadelphia, Pa.; vice-president for the south, J. P. Gray, Nashville, Tenn.; vice-president for the west, Alfred Owre, Minneapolis, Minn.; corresponding secretary, Burton Lee Thorpe, St. Louis, Mo.; recording secretary, Chas. Butler, Buffalo, N. Y.; treasurer, A. S. Melendy, Knoxville, Tenn. Executive committee, new members—L. Meisenburger, Buffalo, N. Y.; F. B. Kremer, Minneapolis, Minn.; M. F. Finley, Washington, D. C.; Executive council—H. J. Burkhart, chairman, Batavia, N. Y.; J. Y. Crawford, Nashville, Tenn.; A. H. Peck, Chicago, Ills.; F. O. Hetrick, Ottawa, Kan.; B. Holly Smith, Baltimore, Md. Next place of meeting, Boston, 1908.

